# The Base Excision Repair Pathway in the Nematode *Caenorhabditis elegans*

**DOI:** 10.3389/fcell.2020.598860

**Published:** 2020-12-03

**Authors:** Noha Elsakrmy, Qiu-Mei Zhang-Akiyama, Dindial Ramotar

**Affiliations:** ^1^Division of Biological and Biomedical Sciences, College of Health and Life Sciences, Hamad Bin Khalifa University, Education City, Qatar; ^2^Laboratory of Stress Response Biology, Graduate School of Science, Kyoto University, Kyoto, Japan

**Keywords:** *C. elegans*, base excision repair pathway, DNA glycosylases and AP endonucleases, germ cells, survival, phenotypes, DNA damaging agents

## Abstract

Exogenous and endogenous damage to the DNA is inevitable. Several DNA repair pathways including base excision, nucleotide excision, mismatch, homologous and non-homologous recombinations are conserved across all organisms to faithfully maintain the integrity of the genome. The base excision repair (BER) pathway functions to repair single-base DNA lesions and during the process creates the premutagenic apurinic/apyrimidinic (AP) sites. In this review, we discuss the components of the BER pathway in the nematode *Caenorhabditis elegans* and delineate the different phenotypes caused by the deletion or the knockdown of the respective DNA repair gene, as well as the implications. To date, two DNA glycosylases have been identified in *C. elegans*, the monofunctional uracil DNA glycosylase-1 (UNG-1) and the bifunctional endonuclease III-1 (NTH-1) with associated AP lyase activity. In addition, the animal possesses two AP endonucleases belonging to the exonuclease-3 and endonuclease IV families and in *C. elegans* these enzymes are called EXO-3 and APN-1, respectively. In mammalian cells, the DNA polymerase, Pol beta, that is required to reinsert the correct bases for DNA repair synthesis is not found in the genome of *C. elegans* and the evidence indicates that this role could be substituted by DNA polymerase theta (POLQ), which is known to perform a function in the microhomology-mediated end-joining pathway in human cells. The phenotypes observed by the *C. elegans* mutant strains of the BER pathway raised many challenging questions including the possibility that the DNA glycosylases may have broader functional roles, as discuss in this review.

## Introduction

**FIGURE 1:**
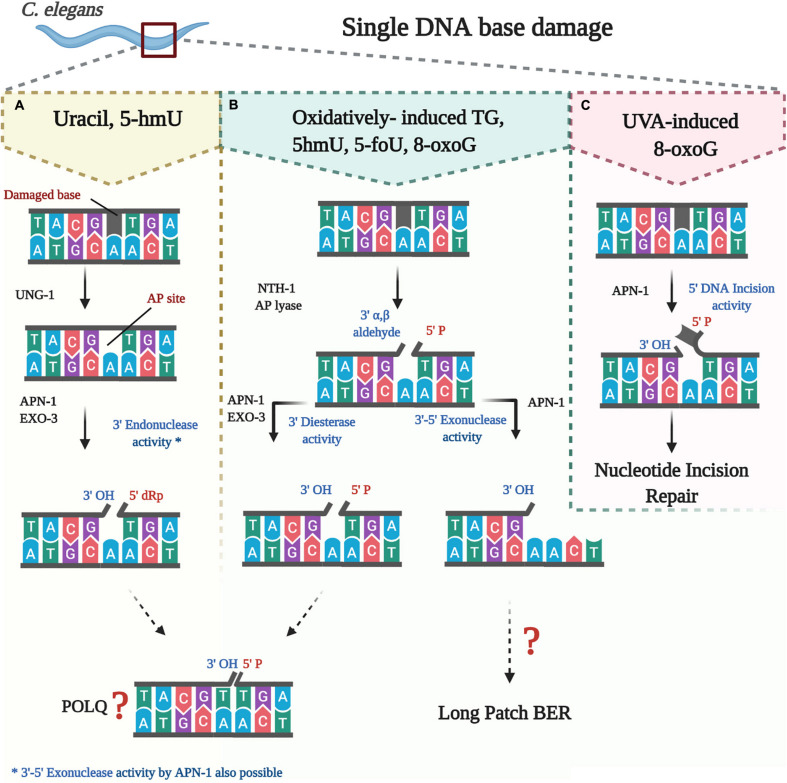
Processing of oxidatively damaged bases by the BER pathway in *C. elegans*. **(A)** uracil and likely 5-hmU are removed by UNG-1 to create an AP site that is processed by either APN-1 or EXO-3 or both. **(B)** oxidatively damaged bases are removed by NTH-1, which then nicks the resulting AP site to create a blocked 3 end that needs further processing by either APN-1 or EXO-3 or both. **(C)** direct nicking of oxidatively damaged base by APN-1.

Damage to the DNA is an inevitable process occurring persistently within a living cell. Various exogenous and endogenous insults induce oxidation, deamination, alkylation, depurination, and other premutagenic changes on the DNA bases that threaten the integrity of the genome. Oxidative base damage alone is estimated to be 10,000 per human cell per day (Lindahl and Barnes, 2000). Several DNA repair mechanisms such as the base excision repair (BER), nucleotide excision repair (NER), mismatch excision repair, and recombinational DNA repair have evolved to function faithfully to evade the toxic and mutagenic consequences of various DNA lesions. The BER pathway primarily repairs endogenously generated DNA lesions and it is. initiated by a DNA glycosylase recognizing the damaged base (Figure 1; Krokan and Bjørås, 2013). Although most DNA glycosylases are substrate-specific, some display redundancy and broad substrate specificity (Katafuchi et al., 2004). For instance, of the eleven BER DNA glycosylases conserved in humans, four are able to recognize uracil bases in the DNA, which can arise through cytosine deamination or as a result of misincorporation by a DNA polymerase (Whitaker et al., 2017). Once an aberrant base is recognized by a monofunctional DNA glycosylase, the enzyme cleaves the N-glycosidic bond between the base and the deoxyribose sugar. This creates an apurinic/apyrimidinic (AP) or also refer to as an abasic site. These non-instructional AP sites are premutagenic as they can lead to the misincorporation of the incorrect base by DNA polymerases, and thus must be removed by an AP endonuclease. The AP endonuclease cleaves the DNA backbone 5′ to the AP site, leaving behind a 3′-hydroxyl and a 5′-deoxyribophosphate (dRP) residue. The gap created by the removal of the damaged base is filled by the single insertion of the correct nucleotide through the action of a DNA polymerase. The same DNA polymerase has a deoxyribophosphodiesterase activity that removes the 5′-dRP, enabling DNA ligase to enter and seal the nick to complete the pathway that is referred to as “Short Patch” BER. In mammalian cells, the ligation step requires the formation of a complex that includes DNA ligase I or III and the scaffolding protein X-ray Repair Cross-Complementing protein 1 (XRCC1) (Tomkinson et al., 2001).

Processing of the damaged base can be initiated by a bifunctional, instead of a monofunctional DNA glycosylase. In addition to the N-glycosylic activity, bifunctional DNA glycosylases such as endonuclease III (NTH) are capable of also cleaving the DNA backbone 3′ to the AP site in a β-elimination reaction. This creates a 5′-phosphate group and a 3′-blocking group such as the 3′-α, β-unsaturated aldehyde residue created by NTH1. This 3′-blocking group must be removed to allow the insertion of the new nucleotide. The 3′-blocking group is removed by the 3′-phosphodiesterase activity that is endowed by AP endonucleases, thereby generating a 3′-hydroxyl group to enable nucleotide insertion and ligation by DNA polymerase and DNA ligase, respectively. It is believed that when the BER pathway is initiated by a bifunctional DNA glycosylase to remove oxidatively damaged bases, the “Long Patch” BER serves as an alternative repair process (Klungland and Lindahl, 1997). In the Long Patch BER a stretch of several nucleotides, often 2–15, is inserted and at the same time the 5′-end is displaced by the newly synthesized complementary strand. The displaced strand forms what is known as a 5′-flap, which is then removed by a Flap structure specific endonuclease FEN-1, a specialized nuclease that removes the 5′-overhanging flap, thus preparing the nick to be sealed by a DNA ligase. Apparently, directing the cell machinery toward Long Patch BER depends on several factors such as existence of cluster base damage, the abundance of certain replication factors including the proliferating cell nuclear antigen, as well as the interaction with downstream enzymes such as polymerase β or the flap endonuclease (Klungland and Lindahl, 1997).

Base excision repair plays an essential role in maintaining the integrity of the genome and proper functioning of cells. Defects in BER are linked to the development of several diseases [reviewed in Wallace et al. (2012) and Wallace (2014)]. For instance, mutations in polymerase β are found in nearly 30% of human cancers (Starcevic et al., 2004), whereas mutations in UNG-1 have been linked to colorectal cancer and glioblastoma (Moon et al., 1998; Broderick et al., 2006) and recently, mutations in XRCC-1 have been linked to the development of hepatocellular carcinoma (Mattar et al., 2018). Moreover, mutations in BER have been linked to neurodegenerative diseases such as Alzheimer’s and Parkinson’s diseases (Kwiatkowski et al., 2016; Sanders et al., 2017), as well as the established contribution of a deteriorating BER system to the process of aging (Vijg and Suh, 2013). Thus, it is of essence that researchers delineate the components of the BER pathway and its interaction with genomic lesions and other cellular components.

The redundancy of DNA glycosylases, as well as the essentiality of downstream enzymes in early life stages have made it challenging to create nullizygous animal models that are viable and to demonstrate a clear phenotype. *Caenorhabditis elegans* is a powerful animal model for studying base excision repair for several reasons. While it has a short life span, high number of progenies and convenient for growth in the laboratory, it possesses fewer BER enzymes exhibiting minimal redundancy to explore the specific phenotypic effects of single BER gene. In addition, many viable nullizygous knockout and knockdown of *C. elegans* BER genes are available or can be created to study this pathway. In this review, we aim to discuss the components of BER and the resulting mutant phenotypes in *C. elegans*. We will highlight the gaps in understanding the BER system in *C. elegans* and to tackle the longstanding question as to why this organism has only two DNA glycosylases, while several of these enzymes can be found in bacteria, yeast and human cells.

## First Evidence of BER in *C. elegans*

The use of *C. elegans* as a model for studying DNA repair first captured the attention of scientists in the late 1990’s – early 2000’s when *C. elegans* was shown to resist irradiation-induced DNA damage (Chin and Villeneuve, 2001). Later, the existence of a BER pathway in *C. elegans* was first reported when two AP endonuclease genes *apn-1* and *exo-3* were isolated from *C. elegans* and the predicted amino acid sequences were shown to share nearly 40% identity with the functionally established counterparts from *Saccharomyces cerevisiae*, Apn1 and Apn2, and *Escherichia coli*, Nfo (endonuclease IV) and Xth (exonuclease III), respectively (Masson et al., 1996; Shatilla et al., 2005b). Subsequent studies defined that APN-1 and EXO-3 from *C. elegans* were indeed AP endonucleases (Shatilla et al., 2005a; Yang et al., 2012). As further discoveries came along that include the identification of two DNA glycosylases, UNG-1 and NTH-1, *C. elegans* formed a unique model for the study of BER as it became clear from rigorous homology searches and biochemical analyses that this organism possesses fewer enzymes as compared to the human complements of BER enzymes. This made *C. elegans* a simpler model to study the much more complex system in humans. In addition, identifying phenotype of DNA-repair deficient worms, forms a plausible solution to uncover corresponding phenotype in humans, which is often masked by the overlapping activity of the different enzymes constituting the BER pathway. Figure 1 summarizes the BER pathway in *C. elegans*.

## DNA Glycosylases in *C. elegans*

The first DNA glycosylase to be discovered was a uracil DNA glycosylase of *E. coli* in the laboratory of Tomas Lindahl in 1974, setting the stage to show that a DNA glycosylase initiates the BER pathway by recognizing the uracil base as a lesion in the DNA (Friedberg and Lindahl, 2004). This seminal work was followed by many discoveries establishing that DNA glycosylases are well defined across several organisms (Denver et al., 2003; Wallace, 2014). The *C. elegans* uracil DNA glycosylase was initially described in early 2000’s using an *in vitro* BER repair assay, which monitored the removal of an installed uracil opposite guanine (U**⋅**G) in a 42-mer double stranded oligonucleotide substrate (Shatilla and Ramotar, 2002). Several years later, the *ung-1* and *nth*-1 genes encoding Uracil DNA Glycosylase UNG-1 and Endonuclease III NTH-1, respectively, were identified and characterized in *C. elegans* (Nakamura et al., 2008; Morinaga et al., 2009). Despite several attempts, including extensive homology searches and most importantly enzymatic assays designed to remove specific DNA lesions such as 8-oxoGuanine, no other DNA glycosylases have been found in *C. elegans* besides UNG-1 and NTH-1 (Denver et al., 2003; Papaluca et al., 2018). It seems puzzling that this multicellular organism conserved only two DNA glycosylases when the unicellular *E. coli*, the budding yeast *S. cerevisiae* and human cells have conserved eight, five and eleven DNA glycosylases, respectively (Denver et al., 2003; Wallace, 2014). Assuming that there are indeed only two DNA glycosylases in *C. elegans*, this raises an important question of the substrate specificity and the multifunctionality of CeUNG-1 and CeNTH-1, as compared to other organisms and making *C. elegans* an interesting model for the study of BER pathway.

### The Enzymatic Activities of *C. elegans* UNG-1 and NTH-1

Uracil in DNA arises *in vivo* through the incorporation of dUTP opposite adenine by DNA polymerases during DNA replication and via spontaneous deamination of cytosine to give rise to a U**⋅**G mispair that leads to a C**⋅**T transition mutation (Tye et al., 1978; Friedberg and Lindahl, 2004). In humans, four enzymes are capable of recognizing mispaired uracil bases and initiate its excision repair. These enzymes are Uracil DNA glycosylase (UNG), single-strand-selective monofunctional uracil glycosylase 1 (SMUG-1), thymine DNA glycosylase (TDG), and methyl CpG binding domain protein 4 (MBD4). UNG removes uracil from both double and single stranded DNA, as well as possesses the ability to process other substrates such as 5-fluorouracil (5-FU) and cytosine oxidation products including alloxane, isodialuric acid, and 5′, 6′-dihydrouracil (Dizdaroglu et al., 1996; Fischer et al., 2007). SMUG-1, not only excises U, but it is also capable of processing other uracil derivatives such as 5-hydroxyuracil (5-hU) and 5-hydroxymethyl uracil (5-hmU), which can arise as a result of oxidation of thymine. TDG, on the other hand, removes T, U, and 5-hmU when mispaired with G in double stranded DNA. Similarly, the glycosylase domain of MBD4 functions to remove T, U, and 5-hmU opposite a G, but mainly in CpG-rich regions of the DNA.

The genome of *C. elegans*, however, encodes only a homologous sequence to the human uracil DNA glycosylase with 58.2% similarity (Nakamura et al., 2008); none of the other three enzymes (SMUG-1, TDG, and MBD4) appear to be encoded by the organism (Denver et al., 2003; Nakamura et al., 2008). In 2008, using BLAST search Nakamura et al. (2008) were able to identify the sequence of CeUNG-1, consistent with the initial observation made in 2000 for the presence of UNG-1 in *C. elegans*. The authors reported that UNG-1 is a homolog of the *E. coli ung* gene product, with 49% shared identity. *C. elegans ung-1* is located on chromosome III, containing three exons, and its product is ∼ 32 kDa in size (Nakamura et al., 2008). Similar to DNA glycosylases present in *E. coli* and mammalian cells, the structure of CeUNG-1 contains two active sites A and B, where the former is present between residues 116 and 137 and the latter is present between residues 247 and 253 (Pearl, 2000; Denver et al., 2003; Nakamura et al., 2008). Cloning of *C. elegans ung-1* into *E. coli* strain BL21 enabled the purification of UNG-1. The glycosylase demonstrated efficient removal of U**⋅**A and U**⋅**G mismatches from dsDNA, although with a stronger affinity toward the U**⋅**G mismatched base pairs (Nakamura et al., 2008). This is confirmed in another study, where UNG-1 demonstrated two-fold increase in processing of U**⋅**G lesions (Skjeldam et al., 2010). CeUNG-1 can also remove uracil from ssDNA (Nakamura et al., 2008; Skjeldam et al., 2010). Inhibition of CeUNG-1 by the *lactobacillus* uracil glycosylase inhibitor Ugi strongly supported that UNG-1 belongs to the UNG family 1 group (Karran et al., 1981; Nakamura et al., 2008; Skjeldam et al., 2010).

Recently, it has been reported that UNG-1 may possess the ability to process the oxidized base lesion 5-hmU. This is based on phenotypic analysis of *C. elegans ung-1* mutant showing decreased survival upon exposure to the nucleoside form of 5-hmU (Papaluca et al., 2018). We propose that *C. elegans* UNG-1, unlike Ung from other organisms, may have evolved to acquire a broader substrate specificity and thus could act as the dominant DNA glycosylase *in vivo* to remove various modified forms of uracil such as the 5-hmU lesion. Because *C. elegans* lacks the related human SMUG1 DNA glycosylase, which has been shown to remove 5-hmU (Boorstein et al., 2001), supports the notion that UNG-1 may have a role in 5hmU removal. *C. elegans* UNG-1 shares a modest 12.6% identity with SMUG1 (Zauri et al., 2015). A closer examination of the identity revealed that *C. elegans* UNG-1 shares five amino acid residues Ser58, Pro218, Gly226, Glu233, and Leu234 that are unique to human SMUG1 residues Ser48, Pro166, Gly174, Glu181 and Leu182 and which are absent in human UNG1 (Papaluca et al., 2018). Whether these five amino acid residues are involved in conferring upon *C. elegans* UNG-1 the ability to recognize and process 5-hmU in a manner similar to human SMUG1 will need to be investigated.

The second DNA glycosylase found to initiate BER pathway in *C. elegans* is NTH-1. NTH-1 belong to the helix-hairpin-helix (HhH) superfamily of DNA glycosylases (Endonuclease III, Nth) that are conserved in almost all forms of life (Denver et al., 2003). Members of the NTH-1 family share enzymatic activities that recognize and remove oxidatively damaged pyrimidine bases such as thymine glycol (Tg), 5-formyluracil (5-foU), and 5-hmU in DNA. Unlike the monofunctional UNG-1, NTH-1 is a dual function enzyme that has an AP lyase in addition to its DNA glycosylase activity. Following removal of the oxidized damage pyrimidine base, the resulting AP site left by NTH-1 can be cleaved by its AP lyase activity. NTH-1 cuts the DNA backbone 3′- to the AP site in a β-elimination reaction, and generates a 3′-α, β-unsaturated aldehyde group that is removed by the 3′-diesterase activity of either APN-1 or EXO-3 to create a 3′-OH allowing for nucleotide insertion by DNA polymerase (Figure 1; Mazumder et al., 1991; Shatilla et al., 2005a; Yang et al., 2012).

It is noteworthy that although the major domain is well conserved amongst the family of Nth proteins, the sequences of amino acid in the N-terminal region are different between CeNTH-1 and *E. coli* Nth-1 (Morinaga et al., 2009). This is reminiscent of the long N-terminal found in *C. elegans* APN-1, but not *E. coli* Nfo (see below). Interestingly, expressing only the portion of *C. elegans nth-1* sequence reported in phylogenetic studies as the NTH-1 ortholog in *E. coli* produced a protein that is unable to perform NTH-1-like activities (Morinaga et al., 2009). However, modification of the *nth-1* sequence by inclusion of the 117 bp corresponding to the missing N-terminal restored the DNA glycosylase activity (Morinaga et al., 2009). This full length CeNTH-1 shared 67.4% similarity to the human NTH-1, and catalyzed the removal of oxidized pyrimidine bases, particularly thymine glycol (Tg), 5-formyl uracil (5-foU), 5-hmU, and to a weaker extent, 8-oxoguanine (8-oxoG) (Morinaga et al., 2009). *In vivo* measurement of DNA repair revealed that NTH-1 is capable of restoring nuclear DNA integrity within 24 h of exposure to the oxidizing agent H_2_O_2_ that creates various oxidized bases such as thymine glycol (Hunter et al., 2012). Repair in mitochondrial genome is also seen, although less efficiently (Hunter et al., 2012). The most compelling evidence that NTH-1 functions as a DNA glycosylase *in vivo* came from a study whereby the *C. elegans nth-1* gene was expressed in the *E. coli* double mutant strain deficient for both Nth and Nei; the two DNA glycosylases known to repair oxidized pyrimidine bases in the bacterium. Expression of the *C. elegans nth-1* gene in the *E. coli nth;nei* double mutant strain rescued it from the genotoxic effects of H_2_O_2_, consistent with a direct role for NTH-1 in the repair of oxidative DNA lesions (Morinaga et al., 2009).

### Phenotype of *ung-1* and *nth-1* Deficient *C. elegans*

**FIGURE 2:**
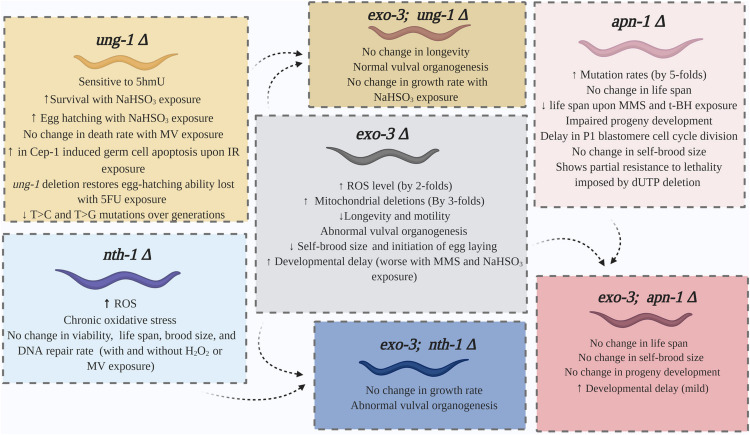
Summary of the phenotypes displayed by the single and double mutants of the BER pathway of *C. elegans*.

The phenotypic and physiologic consequences of *C. elegans ung-1* knockdown were assessed in several reports (Figure 2; Nakamura et al., 2008; Skjeldam et al., 2010; Papaluca et al., 2018). Despite deletion of the *ung-1* gene, the resulting mutant strain demonstrated a normal egg-laying rate and larval development, in addition to a normal life span compared to N2 strain (Nakamura et al., 2008). Interestingly, the *C. elegans ung-1* mutant was no more sensitive than the wild type (WT) upon exposure to sodium hydrogen sulfite (NaHSO_3_); a DNA damaging agent that induces cytosine base deamination and creates U**⋅**G lesion (Burgers and Klein, 1986; Nakamura et al., 2008; Miyaji et al., 2018). These findings are unique to *C. elegans* as *S. cerevisiae* and *E. coli* cells lacking Ung activity showed high sensitivity to NaHSO_3_ (Simmons and Friedberg, 1979; Burgers and Klein, 1986). One possibility for the lack of sensitivity of the *C. elegans ung-1* mutant toward NaHSO_3_ could be due to efficient bypass of the U**⋅**G lesion, which may not occur in *E. coli* or yeast *ung1* mutants. As such, we postulate that the deficiency of UNG-1 may lead to the recruitment of a translesion polymerase to bypass the U**⋅**G lesion, thereby avoiding toxic AP sites and single strand breaks that would be ordinarily created by the presence of UNG-1 and the subsequent processing by an AP endonuclease.

Recently, UNG-1 was implicated to process the thymine oxidation product 5-hmU (Papaluca et al., 2018). This was detected through *in vivo* survival analysis where u*ng-1* mutant worms were shown to be sensitive to the genotoxic effects of 5-hmU, but not the *nth-1* mutant strain, indicating that UNG-1 could be the main DNA glycosylase to process 5-hmU bases in the nematode genome. This observation is also consistent with a previous finding that the germ cells of *ung-1* mutants might be sensitive to ionizing radiation underscoring the role of UNG-1 in repairing oxidized DNA lesions induced by this agent (Skjeldam et al., 2010). If indeed the above observations are correct, then it would be the first time a substrate other than uracil has been postulated for UNG-1 in *C. elegans.*

**FIGURE 3:**
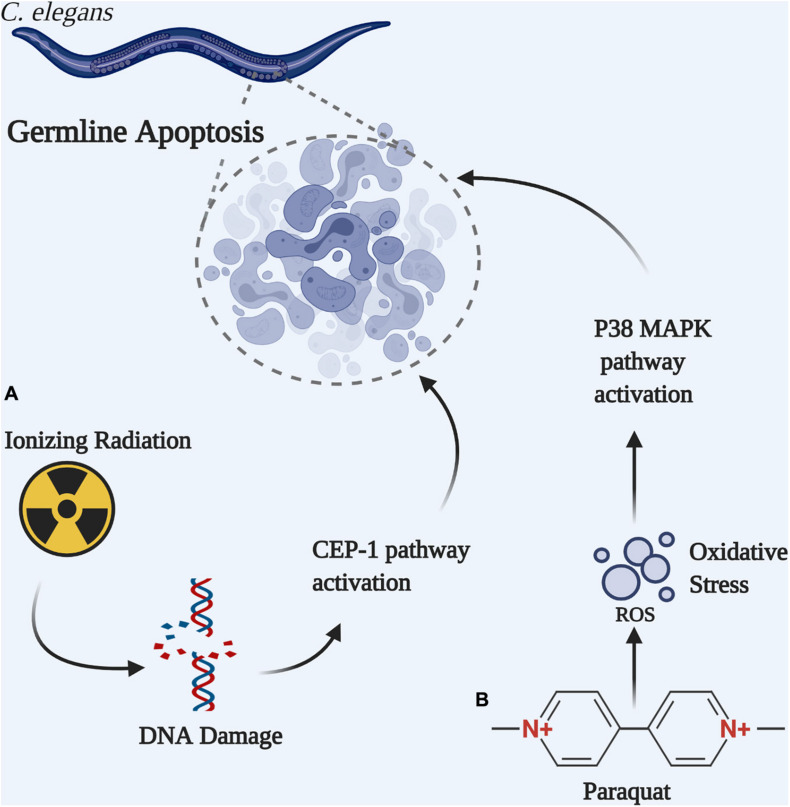
Germline apoptosis can be initiated by different pathways in *C. elegans*. **(A)** Ionizing radiation-induced DNA double strand break activates the CEP-1-dependent apoptotic pathway. **(B)** Paraquat-induced oxidative DNA damage activates the P38 MAPK-dependent apoptotic pathway.

In *C. elegans*, germ cell apoptosis is a natural and very sensitive response to excessive endogenous or exogenous DNA damage, as opposed to monitoring progeny survival. Different genotoxic agents induce apoptosis through different pathways (Figure 3). For example, ionizing radiation provokes oxidative DNA damage in a CEP-1 dependent apoptotic pathway in wild type *C. elegans* (Hoffman et al., 2014). CEP-1, the *C. elegans* ortholog of the mammalian proapoptotic transcription factor p53, is required to induce CED-1 that forms a ring around apoptotic cells and is easily visualized as CED-1:GFP (Hoffman et al., 2014; Papaluca et al., 2018). In contrast, the oxidant paraquat (Methyl Viologen) induces germline apoptosis through a different mechanism that involves the p38 MAPK-dependent pathway (Salinas et al., 2006). Congruent to these reports, Skjeldam et al. (2010) observed that CEP-1-dependent apoptotic pathway is not induced by paraquat treatment in the wild-type, nor in the *ung-1* deficient worms. Furthermore, the authors showed that CEP-1 induced germ cell apoptosis increased in *ung-1* deficient nematode compared with the wild-type N2 after exposure to ionizing radiation and thus established a role for UNG-1 in DNA repair following exposure to ionizing radiation (Skjeldam et al., 2010). Upon exposure to paraquat, cell death increased in the wild-type worms, but not in the *ung-1* deficient worms. This unexpected finding is due to alteration in transcriptional regulation where it has been observed that the p38 MAPK pathway and other response pathways to oxidative stress have been suppressed in the *ung-1* deficient nematode thereby impairing apoptosis (Skjeldam et al., 2010).

Studies for identification of phenotypic changes in *nth-1* deficient *C. elegans* were also conducted. As indicated in Figure 2, *nth-1* mutants show normal life span, embryonic viability, and brood size (Morinaga et al., 2009; Hunter et al., 2012; Kassahun et al., 2018). These phenotypes were not affected by exposure to H_2_O_2_ or paraquat (Morinaga et al., 2009). Similar to the wild type strain N2, *nth-1* deficient strain also exhibits effective repair of its nuclear and mitochondrial DNA (mtDNA) upon exposure to H_2_O_2_ (Hunter et al., 2012; Volkova et al., 2020). However, these *nth-1* knockout strains compared to wild type do undergo chronic oxidative stress and mitochondrial dysfunction, as reflected by elevated steady state levels of ROS (Kassahun et al., 2018). This is congruent with an observed transcriptional upregulation of cellular proteins involved in activation of SKN-1 (Fensgard et al., 2010; Kassahun et al., 2018) a transcription factor that mediates transcriptional responses to oxidative stress (Kahn et al., 2008). Downregulation of insulin/IGF-1 (IIS) pathway is also seen in *nth-1* mutants (Fensgard et al., 2010). Apparently, suppression of the IIS pathway is shown to induce longevity in *C. elegans* (Rusyn et al., 2007). These alterations could represent a defense mechanism against oxidative DNA damage and explain the normal lifespan seen in NTH-1 deficient strain.

It is interesting to note that both UNG-1 and NTH-1 deficient mutants show a similar morphology of impaired CEP-1 independent apoptotic response to oxidative damage, although NTH-1 is the main DNA glycosylase involved in oxidative damage repair in BER pathway (Skjeldam et al., 2010; Kassahun et al., 2018). This has been explained by the identification of compensatory transcriptional changes that attenuate p38-MAPK-stress activated pathways, and not due to loss of DNA repair from the deleted DNA glycosylase (Fensgard et al., 2010; Skjeldam et al., 2010; Kassahun et al., 2018).

## AP Endonucleases in *C. elegans*

Following the initiation of base excision repair by a monofunctional DNA glycosylase to generate an AP site or by a bifunctional DNA glycosylase that subsequently creates 3′-blocking group, an AP endonuclease is recruited to process the secondary lesion to produce a 3′-hydroxyl group for DNA repair synthesis. AP endonuclease enzymes are multifunctional proteins that are conserved in all organisms (Li and Wilson, 2014). In addition to their endonuclease function to cleave AP sites, AP endonucleases possess 3′-diesterase activity, 3′-5′-exonuclease activity, as well as 5′-DNA incision activity that contributes to nucleotide incision repair (Hosfield et al., 1999; Daley et al., 2010; Redrejo-Rodriguez et al., 2016). The AP endonucleases belong to one of two families, the exonuclease III family (Exo III) or the endonuclease IV (Endo IV) family. While both families are metal-dependent, a distinct feature of Endo IV enzymes is the presence of tightly bound Zn^+2^ ions to perform cleavage of the phosphodiester bond, while EXO III enzymes require the addition of Mg^+2^ ions to exert its functions (Hosfield et al., 1999; Daley et al., 2010; Redrejo-Rodriguez et al., 2016).

It is important to highlight that in human cells two AP endonucleases have been identified, APE1 and APE2. APE-1 is the major AP endonuclease as it is responsible for about 95% of DNA repair activity (Demple et al., 1991; Li and Wilson, 2014). APE-1, sometimes referred to as redox effector factor-1 (Ref-1), is capable of performing other cellular functions related to redox regulation of ubiquitous and tissue specific transcription factors (Kelley et al., 2012). In humans, inappropriate APE1 expression or function are associated with several cancerous and neurodegenerative diseases (Xanthoudakis et al., 1996; Whitaker et al., 2017). This renders APE1 an attractive target for the diagnosis and treatment of cancer as well as other diseases (Park et al., 2014; Ayyildiz et al., 2020). Dual deletion of the *APE1* alleles in mice causes early embryonic lethality that led to a set of challenging experiments to study the enzyme-phenotype correlations. Because of the conserved enzymatic functions as well as the viability of *apn-1* or *exo-3* null mutants, attracted the interest of many scientists to use *C. elegans* as a suitable animal model for the study of AP endonuclease enzymes in BER and to deduce possible impact on health and disease.

### Structural and Functional Analysis of *C. elegans* EXO-3 and APN-1

The first report of an AP endonuclease existing in *C. elegans* was published in 1996 (Masson et al., 1996). The gene identified through cDNA library screening encoded a 30 kDa protein with 278 amino acids that shared homology with *E. coli* Endo IV and was called CeAPN-1. Expression of CeAPN-1 in *E. coli* mutants deficient in AP endonuclease activities to test for cross-species complementation failed to produce a functional protein, as the protein was degraded (Masson et al., 1996). However, it was confirmed that an active AP endonuclease activity was present in crude extracts of *C. elegans*, but the activity could not be attributed exclusively to the identified CeAPN-1 (Masson et al., 1996). Further investigations revealed that embryonic extract derived from *C. elegans* contained an AP endonuclease activity with high similarity to that displayed by the *E. coli* exonuclease III (Xth), particularly in its resultant nicked product and in addition to its Mg^+2^-dependence (Shatilla and Ramotar, 2002). This finding raised the possibility there must be at least two AP endonuclease genes in *C. elegans*, *Ceapn-1*, and *Ceexo-3*. Indeed, a *C. elegans* exonuclease-3 (EXO-3) was later identified by using cross-species complementation analysis whereby a mutant *S. cerevisiae* strain YW778, lacking three genes including *APN1* and *APN2* encoding the two AP endonucleases, was exploited to isolate the *C. elegans* AP endonuclease genes (Shatilla et al., 2005a,b). The YW778 mutant strain is sensitive to DNA damaging agents such as MMS that induces formation of AP sites (Vance and Wilson, 2001). Expression of *C. elegans* APN-1 or EXO-3 successfully rescued the yeast DNA repair capabilities and recovered its resistance to MMS (Shatilla et al., 2005b).

Exonuclease-3 of *C. elegans* shares 44% and 64% homology with *E. coli* Xth (exo III) and human APE1, respectively (Shatilla et al., 2005b). *In vitro* DNA repair assays demonstrate that EXO-3 is a strong AP endonuclease that cleaves AP sites to produce a 3′-OH and 5′-dRp ends in the presence of very low Mg^+2^ concentration (Shatilla et al., 2005b). Addition of 5 mM EDTA, a metal chelating agent, completely abolishes the enzymatic endonuclease activity. EXO-3 also possesses a 3′-diesterase activity capable of excising 3′-α, β-unsaturated aldehyde residues on DNA oligonucleotides that are generated by the action of NTH-1 AP lyase on AP sites. However, it seems this enzymatic activity has a more stringent requirement for Mg^+2^ ions and requires a minimum of 1 mM MgCl_2_ to be added to the *in vitro* reaction mixture for the 3′-diesterase activity to be evident (Shatilla et al., 2005a). Despite its name, *C. elegans* EXO-3 does not display a 3′-5′-exonuclease activity. This is contrary to human APE1 or Xth of *E. coli*, however, the 3′-5′-exonuclease activity is harbored by the *C. elegans* APN-1 instead, in a manner similar to the yeast Apn1 (Vance and Wilson, 2001). *In vitro* assays also failed to detect any activity of a direct 5′-DNA incision toward oxidized bases such as 5,6-dihydroxyuridine, suggesting that *C. elegans* EXO-3 is not directly involved in nucleotide incision repair pathway as human APE1 (Shatilla et al., 2005a; Li and Wilson, 2014). In contrast, *C. elegans* APN-1, and not EXO-3, appears to share more functions with human APE1 (see below) (Yang et al., 2012).

Structural analysis of EXO-3 revealed that substitution of the amino acid His at 279 with Ala or replacing Asp at 190 with Ala completely diminishes the DNA repair capability of EXO-3, and these variants can no longer rescue the AP endonuclease deficient yeast YW778 strain from the genotoxic effects of MMS or agents that create DNA single strand breaks with blocked 3′-ends (Shatilla et al., 2005a). Indeed, *in vitro* experiments with H279A and D190A variants confirm loss of EXO-3 function. Nevertheless, substitution of Glu68 with Ala results in a variant (E68A) that performs AP endonuclease and 3′-diesterase activity *in vitro* in the presence of additional Mg^2+^, but these activities are lost *in vivo*, possibly due to an impaired process beyond the AP endonuclease step (Shatilla et al., 2005a). Interestingly, band-shift experiment revealed that purified native EXO-3 does not stay bound to the AP site substrate in either the absence or the presence of embryonic extract derived from *C. elegans*. In contrast, the purified EXO-3 variant E68A retards the mobility of the AP site substrate only in the presence of the extract. Since purified E68A alone has no effect on the mobility of the AP site substrate, it is assumed that dissociation of the variant from the substrate is a slow process thereby trapping a protein from the extract (Shatilla et al., 2005a). The identity of this protein is under investigation and it is likely a component of the BER pathway.

The second AP endonuclease functioning in the BER pathway in *C. elegans* is APN-1. Although the *apn-1* gene was identified in 1996, the encoded APN-1 protein was only characterized many years later, even after EXO-3, as explained below (Yang et al., 2012). RNA interference studies indicated that knock down of APN-1 caused the animal to lose its resistance to DNA damage induced by MMS (Zakaria et al., 2010). This suggests a unique function for APN-1 that is not substituted by EXO-3 even though both enzymes have the ability to process MMS-induced AP sites (Shatilla and Ramotar, 2002). This prompted the isolation and characterization of APN-1 even though this brought along many new challenges (Yang et al., 2012).

The *apn-1* gene, which is located on chromosome 2 encodes a 396 amino acid-long APN-1 polypeptide (UniProt database) (APN-1, 2020). The APN-1 amino acid sequence from 119 to 396 shared 63% homology with both *S. cerevisiae* Apn1 and *E. coli* Nfo (Shatilla et al., 2005b). The N-terminal amino acid stretch (1–63) of the protein is not directly related to the nuclease activities of the protein, however, this segment is essential for APN-1 localization into the nucleus (Wang et al., 2014). This was evident from an APN-1 variant (1–63Δ) that retained cytoplasmic distribution compared to the nuclear localization of the full-length APN-1 when tested in a yeast system (Wang et al., 2014). Isolation of the *apn-1* gene from a *C. elegans* library designed for yeast two-hybrid screening did not contain the full-length APN-1, and instead carried only a portion of APN-1 from amino acid 119 to 396 (Shatilla et al., 2005b; Wang et al., 2014). Expression of this truncated APN-1 (119–396), which included the region essential for endonuclease activity, to test for cross specie complementation in the yeast AP endonuclease deficient strain YW778 showed no ability to rescue this yeast YW778 mutant strain from MMS-induced AP sites, and thus this strain remained very sensitivity to MMS. Interestingly, the inclusion of a nuclear localization signal to the APN-1 (119–396) caused its localization to the nucleus and which restored full MMS resistance to the YW778 strain to the level of the wild type yeast strain YW465 (Yang et al., 2012; Wang et al., 2014). This approach has allowed the purification and characterization of APN-1, and like yeast Apn1 and *E. coli* endo IV, the CeAPN-1 required no additional metal ions for enzymatic activities. Similar to other endo IV family members, APN-1 of *C. elegans* performs four DNA repair functions with the following enzymatic activities, AP endonuclease, 3′-diesterase, NIR and 3′-5′-exonuclease (Yang et al., 2012). It is noteworthy that the diesterase activity of APN-1 requires relatively higher protein concentrations to be evident, suggesting that APN-1 preferentially cleaves abasic sites to generate a 3′-OH at higher rates than cleaving 3′-blocking groups generated by AP lyases (Yang et al., 2012; Wang et al., 2014). As stated earlier, APN-1 incorporated the 3′-5′-exonuclease activity, which is lacking in the EXO-3 enzyme suggesting that APN-1 could have a broader function in *C. elegans* (Yang et al., 2012; Wang et al., 2014). It is worth noting that CeAPN-1 possesses all the same activity of human APE1, except the ability to serve as a redox factor whereby APE1 has a key cysteine C65 that can reduce a number of transcription factors such as p53 (Nassour et al., 2016). While APE1 is essential in mammalian cells, deletion of either *apn-1* or *exo-3* or both genes in *C. elegans* do not cause lethality, raising the possibility of the importance of the redox function of APE1. So far, no homolog of CeAPN-1 (endo IV member) has been found in human cells, although a second APE1 like enzyme, APE2, with limited function has been isolated and partially characterized (Hadi et al., 2002; Guikema et al., 2007; Burkovics et al., 2009).

Site-directed mutagenesis allowed for the creation of two APN-1 variants, the E261G and the E215G (Yang et al., 2012). Based on the ribbon structure of APN-1 alone and in contact with damaged DNA, it appears that glutamate at position 261 is located within the metal binding pocket and comes in direct contact with the DNA strand, while the glutamate residue at 215 is not directly involved in metal binding (Yang et al., 2012). Although neither substitutions affected the expressed protein size or structure, the variant E215G, and not E261G, was capable of preventing spontaneous DNA mutations as well as rescuing the mutant YW778 strain from bleomycin-induced lesions, suggesting that Glu215 does not play a role in DNA repair (Yang et al., 2012). It appears that replacing the glutamic acid residue at position 261 with a glycine reduced the size of the ion-binding pocket as indicated by the provisional structure of the protein, thus blocking the metal from making contact with the DNA (Yang et al., 2012).

### Phenotypic Changes of EXO3 and APN-1 Mutant Strains

*Caenorhabditis elegans* knockout and knockdown for the APN-1 and EXO-3 AP endonucleases do not lead to inviable phenotypes. The *exo-3* mutant strains often demonstrate a more severe phenotype compared to *apn-1* mutants (Figure 2; Schlotterer et al., 2010; Zakaria et al., 2010; Kato et al., 2015; Miyaji et al., 2018). RNA interference (RNAi) studies indicate that *exo-3* knockdown does not terminate growth completely, rather it results in developmental delay by an average of 6 h from the wild type strain; a delay that is aggravated by exposure to toxic agents such as MMS and NaHSO_3_ (Miyaji et al., 2018). Deletion of *apn-1* in addition to *exo-3* did not alter the observed phenotype as *exo-3;apn-1* double mutants were reported to demonstrate developmental delay as well (Miyaji et al., 2018). In addition, *exo-3* deletion reduced *C. elegans* motility (head and overall body), and induces abnormal vulval organogenesis (Schlotterer et al., 2010; Miyaji et al., 2018). Furthermore, *exo-3* knockdown compromises longevity and reduces life span by an average of 3.1 days, compared to wild type (Schlotterer et al., 2010). Knockout studies conducted later supported this finding, although a wider mean difference of 5.4 days was reported (Kato et al., 2015). This is also accompanied by about 30% reduction of self-brood size than N2 worms (Kato et al., 2015). These impaired phenotypes indicate that *exo-3* is vital to the development of *C. elegans*.

On a cellular level, *exo-3* mutants (RNAi) display 2-fold increase in ROS generation, particularly colocalized with the neuronal system, as well as a 3-fold increase in mtDNA deletions, which is thought to contribute to aging (Harman, 1972; Schlotterer et al., 2010). These effects are thought to be mediated through *cep-1*-dependent mechanism. It appears that wild type *C. elegans* strain with suppressed *cep-1* (RNAi) expression, as well as *cep-1* knockout strain (VC172) show elevated EXO-3 levels (Schlotterer et al., 2010). A similar finding is observed in human cells where p53, the human *cep-1* ortholog, is believed to downregulate the human *exo-3* ortholog APE1 through inhibition of S1 and S1-mediated APE1 transcription (Zaky et al., 2008). However, it is noteworthy that the observed *exo-3* upregulation induced by *cep-1* inhibition may not grant the animal additional resistance to oxidative damage. An RNAi *cep-1* knockout (GK138) strain was shown to be as sensitive as wild type strains to oxidative DNA damage upon placement in a hyperbaric O_2_ chamber (Arum and Johnson, 2007). Although *exo-3* expression levels were not assessed in this particular study nonetheless, the GK138 mutant strain showed an increase in life span that is similar to that observed in wild type *cep-1* RNAi-treated animals created by Schlotterer et al. (2010) which had shown a confirmed increase in *exo-3* levels. Interestingly, knockdown of *cep-1* expression in *exo-3* (RNAi) wild type strains rescued the phenotype with a preserved neuronal function, normalized ROS levels, as well as increased animal motility compared to *exo-3* (RNAi) treated worms alone (Schlotterer et al., 2010). We reasoned that *cep-1* inhibition probably elicits an antioxidant response in the *exo-3* mutant. This can be discerned from the inability to detect changes in oxidative DNA damage levels in *cep-1* mutant NC172 or GK138 strains, as well as a failure to detect a significant change in ROS levels in *cep-1* silenced (RNAi) N2 worms (Arum and Johnson, 2007; Schlotterer et al., 2010). It is likely that EXO-3 may execute other functions that are not DNA repair-related, as in the case of the human APE1 that is involved also in transcription regulation besides a role in BER (Tell et al., 2009).

As in the case of *exo-3* mutants, several distinct phenotypes are observed in *apn-1* knockout and knockdown models. *C. elegans* strains mutated for *apn-1* showed up to 5-fold increase in mutation rates as discerned by a *gfp-lacZ* reporter, which has an insertion that sets *lacZ* out of frame and when mutated brings *lacZ* in frame (Zakaria et al., 2010). Under normal growth conditions, these *apn-1* mutants show no reduction in life span despite the increase burden of mutations (Zakaria et al., 2010; Miyaji et al., 2018). However, upon exposure to DNA damaging agents such as MMS and *tert*-butylhydroperoxide (*t*-BH), the *apn-1* mutants displayed a reduction in life span by 3–4 days (Zakaria et al., 2010). In contrast, exposure of the *apn-1* mutant to UVC radiation did not decrease its lifespan compared to the wild type, implying that APN-1 is not essential for mitigating the genotoxicity of ultraviolet radiation as it is for MMS and *t*-BH (Zakaria et al., 2010). Diminishing APN-1 activity in *C. elegans* is also accompanied by an impaired progeny development. Compared to wild type, *apn-1* mutants (RNAi) show a striking difference of 30% lower egg-hatching rate at 24 h of incubation. The difference in egg-hatching rate is significantly minimized to 10% at 36 h and beyond, indicating an impaired developmental rate in *apn-1* strains compared to wild type (Zakaria et al., 2010). It appears that *apn-1* deletion impacts progeny development at the very early stages of embryogenesis, likely due to the accumulation of lethal mutations in the germ cells.

The first cell to give rise to *C. elegans* is P0 cell which divides into an AB cell and a P1 cell (Brauchle et al., 2003). While AB cell is not affected by DNA damage occurring during this stage, the P1 cell division decreased in response to a DNA damage-activated checkpoint signaling pathway (Brauchle et al., 2003). Time-lapse microscopy revealed that *apn-1* knockdown strains exhibited a delayed P1 cell cycle division by about 39 s (*P* value < 0.003) compared to wild type strains (Zakaria et al., 2010). This delay was specific to P1 cells as no difference in AB cell cycle length was noted between wild type and *apn-1* mutated cells (Zakaria et al., 2010). Nevertheless, impairment of progeny development in *apn-1* mutants was not necessarily associated with altered progeny production; *apn-1* mutants laid eggs in a similar pattern to that of wild type, as evident in an unaffected self-brood size (Zakaria et al., 2010; Kato et al., 2015). Lastly, APN-1 appears to contribute significantly to the animal lethality resulting from knockdown of the *dut-1* gene encoding the deoxyuridine 5′-triphosphate nucleotidohydrolase (DUT-1). DUT-1 converts dUTP into dUMP, which is the precursor for the synthesis of dTTP, and in the absence of DUT-1 there is an increased in dUTP levels that can be used efficiently by DNA polymerase for incorporation opposite adenine in the genome. DUT-1 inhibition increased uracil incorporation in the DNA and following removal by UNG-1 resulted in an increased level of toxic AP sites that diminished *C. elegans* survival (Dengg et al., 2006). Downregulation of *apn-1* (RNAi) in the *dut-1* mutant partially resolved the imposed lethality as evident by nearly 7% of broods reaching adulthood, in contrast to *exo-3* mutants where only 0.2% successfully reached adult stages (Zakaria et al., 2010). One interpretation of these findings is that UNG-1 removal of uracil is followed by recruitment of APN-1, and not EXO-3, to process the AP sites to create an accumulation of toxic single strand DNA breaks that could further lead to double strand breaks (Zakaria et al., 2010). Under this condition, the downregulation of *apn-1* may not be efficient to suppress the cleavage of the AP sites left by UNG-1. Alternatively, we cannot exclude the possibility that the AP sites left by UNG-1 action are rapidly cleaved *via* competition by the AP lyase activity of NTH-1 to create toxic DNA single strand breaks with 3′-blocked ends (Papaluca et al., 2018). As such, it would be important to test whether knockdown of *nth-1* will completely rescue the lethality caused by *dut-1* mutation in *C. elegans*. In the case of *E. coli* or yeast, the *dut1* or *DUT1* gene, respectively, is essential and an allele of *dut1-1* with compromised activity in yeast is lethal in the absence of *APN1* (Guillet et al., 2006). It is possible that the lethality of the *dut1-1*; *apn1* double mutant may be the result of AP lyases, such as Ntg1 and Ntg2, acting on the accumulated AP sites to generate toxic single strand breaks with 3′-blocked ends. In both model systems, *C. elegans* and yeast, it seems that processing of *dut1*-mediated AP sites by AP lyases may lead to lethality due to accumulation of toxic DNA single strand breaks.

### AP Endonucleases During Life Span of *C. elegans*

The life cycle *of C. elegans* constitutes several stages that can be described as embryogenesis, four larval stages L1-L4, and two adult stages: the young adult, and the gravid adult stage (Altun and Hall, 2009). Articulating findings scattered across several reports of AP endonuclease expression helps draw a general notion of the roles APN-1 and EXO-3 execute throughout *C. elegans* lifespan. *In situ* hybridization of *exo-3* and *apn-1* mRNA identifies that both genes are expressed in the gonads of male and hermaphrodite worms, and that AP sites are efficiently repaired in the gonads of *C. elegans* (Kato et al., 2015). In contrast to *apn-1*, deletion of *exo-3* is accompanied by reduced self-brood size in an *nth-1* dependent manner (∼30% less than the N2 strain) in addition to a delayed initiation of egg-laying, suggesting that exo-3 plays a vital role in the progeny development or gonad maturation phase by processing endogenous lesions that must be processed by NTH-1 and channel to EXO-3 (Kato et al., 2015). During embryogenesis, APN-1 is needed to overcome a DNA damage checkpoint and aid cell cycle progression of the P1 blastomere (Zakaria et al., 2010). Following hatching, expression levels of both APN-1 and EXO-3, as indicated by mRNA transcripts, remains the same throughout the egg and larval stages. Interestingly, at 60 h, corresponding to young adult stage, *apn-1* expression is increased to 2.3 times its levels at 0 h (i.e., egg stage), while to a more extensive level *exo-3* levels increased by 13-fold its levels at the egg stage (Miyaji et al., 2018). Moreover, expression remains at such levels with subsequent progression into the gravid adult stage at 72 h (Miyaji et al., 2018). Upon aging, *exo-3* expression started to decline to reach a level of 45% reduction at 6 days of age, compared to day 1, and remain at such level for the remainder of the nematode life span (Schlotterer et al., 2010). As expected, the declining *exo-3* levels by day 6 correlated with increased mtDNA deletions by day 5. Taken together, there is an essential need for functional AP endonucleases in all of the *C. elegans* life stages, particularly, at adult stages. Moreover, it appears that EXO-3 plays a vital role in the animal development, as evident by a more severe phenotype in *exo-3* mutants compared to *apn-1* (Schlotterer et al., 2010; Zakaria et al., 2010; Kato et al., 2015; Miyaji et al., 2018).

## DNA Polymerase and Ligase in *C. elegans*

### Polymerase Q and Polymerase H

Despite clear evidence of a full BER pathway in *C. elegans*, a homologous sequence for polymerase B (POLB), the X-family DNA polymerase primarily active in base excision repair in human cells was not found (Loeb and Monnat, 2008). Computer-based sequence analysis successfully located 11 putative DNA polymerase sequences, but this approach failed to identify a conserved sequence for POLB or any other X family members (Asagoshi et al., 2012). Furthermore, DNA polymerase assay failed to detect POLB-like activity in the cellular extracts of the nematode (Asagoshi et al., 2012). Notably, several studies suggest that a DNA polymerase theta [or polymerase Q (POLQ) or a polymerase eta polymerase H (POLH)] is involved in DNA repair in *C. elegans*. Both POLQ and POLH belong to the A-family of DNA polymerases and are considered translesion synthesis (TLS) polymerases (also known as bypass polymerases) (Loeb and Monnat, 2008). TLS polymerases catalyze insertion of nucleotides opposite damage site and are error-prone when synthesizing DNA in the absence of a template strand, as in non-homologous end joining (NHEJ) (Loeb and Monnat, 2008). Indeed, POLQ is an essential component of alternative NHEJ in mammalian cells (Mateos-Gomez et al., 2015). Likewise, experiments on *C. elegans* identified that alt-NHEJ was shown to be completely dependent on POLQ, particularly in the germline and is considered the main source of insertion/deletion mutations and thus essential for genetic diversification in *C. elegans* (Roerink et al., 2014; van Schendel et al., 2015). Nonetheless, endeavors to explore a potential role for POLQ in BER pathway led Asagoshi et al. (2012) to identify the presence of a POLQ-like activity in the cellular extracts of *C. elegans* contributing to gap-filling synthesis. Knockout experiments confirmed the involvement of POLQ-1 in the pathway (Asagoshi et al., 2012). More recently, it has been shown that the knockdown of *polq-1* sensitized the animals upon exposure to 5hmU, suggesting a role for POLQ-1 in processing this oxidative DNA lesion via the BER pathway (Papaluca et al., 2018). So far, it would appear that processing, for example, of the oxidized base lesion 5hmU would require the sequential actions of UNG-1, APN-1, and POLQ-1 (Papaluca et al., 2018).

Two additional DNA polymerases, POLH and POLK of *C. elegans* are also implicated in DNA repair (Ohkumo et al., 2006; Akagi et al., 2009; Roerink et al., 2012). Both *C. elegans* POLH and POLK are unique as they do not share homologous recombination repair functions with their human orthologs (Roerink et al., 2012). *polh* and *polk* mutant strains are sensitive to MMS, while the double mutants are even more sensitive to MMS. Since MMS exerts DNA damage through alkylating guanine and adenine bases, which can be repaired by BER, it remains possible that both of these polymerases could participate in the BER pathway in *C. elegans* (Lundin et al., 2005; Roerink et al., 2012).

It remains unclear which of the two possible DNA ligases, LIG-1, and LIG-4, are involved in mending the final step of the BER pathway and which protein would serve the function of coordinating the single strand break repair, if no potential XRCC1 scaffold protein exists in *C. elegans* to orchestrate the recruitments of the various DNA repair proteins in the pathway (Wilson et al., 2017).

### Poly(ADP-ribose)polymerases

Poly(ADP-ribose) polymerases are a group of conserved enzymes involved in apoptotic pathways, post translational modifications, and chromatin remodeling (Hassa and Hottiger, 2008). Notably, PARPs play a role in DNA repair and were proven to be involved in base excision repair (Trucco et al., 1998; Beneke et al., 2000; Le Page et al., 2003). Two homologs sequences were identified in *C. elegans*, namely *pme-1* and *pme-2* (also known as *parp-1* and *parp-2*) (Gagnon et al., 2002). *C. elegans pme-1* encodes a 108 kDa PARP with an N-terminal that contains two Zn^+2^ finger motifs, and a catalytic C-terminal with the canonical PARP motif (Gagnon et al., 2002). PME-1 shares 31% amino acid identity and 78% PARP motif similarity to its human ortholog (Gagnon et al., 2002). PME-2, with a 24% amino acid similarity, is also an active PARP, albeit its shorter primary structure that shared fewer motifs compared to human PARP-2 (Gagnon et al., 2002). Enzymatic inhibition of PME-1 and PME-2 in *C. elegans* was associated with a reduced progeny survival rate after exposure to ionizing radiation, likely due to impaired DNA repair response (Dequen et al., 2005). Knockout worms also demonstrate increased sensitivity to cisplatin treatment reflected in reduced brood size and viability (Crone et al., 2015). Interestingly, BER and transcription-coupled NER were recently shown to be involved in protecting against cisplatin-induced cytotoxicity (Slyskova et al., 2018). Furthermore, *pme-1* and *pme-2* mutants do not experience reduced viability upon exposure to high doses of Manganese (Mn^+2^) (Neumann et al., 2020). This is interesting given that Mn^+2^ exposure was correlated with an impaired poly(ADP-ribosyl)ation-mediated DNA damage response, which is thought to enhance sensitivity to genotoxic drugs (Bornhorst et al., 2010, 2013).

Poly(ADP) ribose polymers are subsequently degraded by poly(ADP-ribose) glycohydrolase (PARG) (St-Laurent and Desnoyers, 2011). *C. elegans* genome encodes two PARGs: *pme-3* and *pme-4*, which share 18% and 22% overall identity, and 42% and 40% PARG motif similarity to human PARG, respectively (St-Laurent et al., 2007). Knockdown (RNAi) of both genes results in enhanced sensitivity to ionizing radiation (St-Laurent et al., 2007), which can be suppressed by an impaired TLS pathway via mutational deactivation of POLQ (Bae et al., 2020). Despite the presence of these enzymes in *C. elegans*, there is no direct evidence to link the PARPs and PARGs to the BER pathway.

## Mutational Signatures in *C. elegans*

Recently, *C. elegans* has been acquiring increasing attention as an animal model for the study of mutational signatures (Meier et al., 2014, 2020a; Volkova et al., 2020). Particularly due to the conservation of key DNA repair pathways, as well as the affordability and feasibility of sequencing successive generations of this short-lived nematode. Genome sequencing of 17 DNA repair deficient mutants indicated that on average, a single mutation per generation is observed not only in wild type, but also in DNA repair defective mutants (Meier et al., 2014; Volkova et al., 2020). With such low mutation rate, animals need to be propagated for at least 20–40 generations to allow rigorous comparison and statistical analysis (Meier et al., 2020a). Generally, base substitutions are the most frequent DNA mutations encountered in WT and mutant strains (Meier et al., 2014). It is interesting that except for MMR mutants, the mutation rates of *C. elegans* DNA repair mutants do not exceed 2 to 5-fold that of baseline (Volkova et al., 2020). BER impaired *exo-3* and *ung-1*, but not *apn-1, pme-1, or pme-2* mutants show a significant increase in base substitution per generation as compared to the WT strain (Meier et al., 2020b). As expected, *ung-1* mutants experience less T > C and T > G mutations (Meier et al., 2014). However, no significant difference in structural variants or insertion/deletion mutations was reported for BER mutants (Meier et al., 2020b). Overall, the maintenance of genomic integrity is surprisingly robust despite knockout of the various DNA repair pathways, accentuating the redundancy of DNA repair mechanisms in *C. elegans* (Volkova et al., 2020).

## BER Enzymes in Relation to Other DNA Repair Pathways

Besides the BER pathway, *C. elegans* also conserved the additional major DNA repair pathways including nucleotide excision, mismatch, non-homologous and homologous recombination pathways. As in the BER pathway, not all the components of the various DNA repair pathways have been found in *C. elegans* when compared to the proteins found in the human DNA repair pathways (Wilson et al., 2017). It is not clear whether *C. elegans* lacks these components, and which are fulfilled by the roles of other unrelated proteins. However, this would require a more in-depth discussion that is not within the scope of this review.

It is noteworthy that *C. elegans* has the ability to recruit components from one DNA repair pathway to execute a function in another pathway. For example, the enzymes of the BER pathway do not necessarily work in isolation, they can show redundancy and overlap with other pathways. In this section, we provide a brief overview of some of the links to other DNA repair pathways.

### Link to MMR

Depletion of *apn-1* (RNAi) in *exo-3* mutant TM4374 strain resulted in increased sensitivity to 5-FU, suggesting that APN-1 is involved in the process that removes 5-FU (SenGupta et al., 2013). Further analysis indicated that 5-FU induced toxicity was not UNG-1 dependent, but EXO-3 and APN-1 dependent, suggesting that an alternative pathway is acting upstream of the AP endonucleases role in nicking of the DNA (SenGupta et al., 2013). Indeed, epistasis analysis indicates that APN-1 and EXO-3 function in cooperation with other Mismatch repair (MMR) pathway enzymes leading to 5-FU induced toxicity. It appears that EXO-3 is required for DNA nicking and subsequent MMR activation, whereas APN-1 induces DNA damage checkpoint activation to allow the repair process (SenGupta et al., 2013).

### Link to NER

As discussed earlier, deletion of *nth-1* does not change the life span of *C. elegans*. On the other hand, if the nucleotide excision repair (NER) gene *xpa-1* is lost, the life span is shortened (Fensgard et al., 2010). The double mutations in the *nth -1* and *xpa-1* genes are expected to further shorten the life span, however, it was reported that the loss of NTH-1 activity restores the life span of short-lived *xpa-1* mutant to the same level as the wild-type worms (Fensgard et al., 2010). Furthermore, the authors have found that depletion of both NTH-1 and XPA-1 induced an oxidative stress response, as well as changes in global expression profiles that involved the upregulation of genes responding to endogenous stress and downregulation of IIS (Fensgard et al., 2010). It is possible that NTH-1 may serve to recognize certain DNA damage that it cannot repair and forming transcription blocking lesion, which may then recruit XPA (Fensgard et al., 2010). Alternatively, NTH-1 may produce toxic single strand breaks requiring XPA-1 function via the NER pathway.

## Perspectives

We initially believed that *C. elegans* would provide a simpler system to study the BER pathway in multicellular organisms, but the findings to date led us to suggest that this model is more complex and raises a number of questions that need to be resolved. The complexity lies in the fact that deleting, for example, the *nth-1* gene triggers a complex regulation of genes to combat oxidative stress thereby dampening any severe phenotypes that might be caused as a consequence of NTH-1 deficiency. Establishing this link might be important to find ways to activate similar systems in human cells to bypass NTH1 defects. Of note, CeNTH-1 has an extended N-terminal that is crucial for the DNA glycosylase activity, although it remains unclear what is the exact function of this region of the enzyme. One tenable possibility is that the N-terminal of NTH-1 might serve to scan the genome and recognize damaged bases to activate the DNA glycosylase activity.

Besides the above challenges, several additional aspects remain unresolved regarding the BER system in *C. elegans*, for example (i) it is unclear whether a second uracil DNA glycosylase exists in *C. elegans* as raised by earlier reports While extracts derived from UNG-1 deficient strain suggest the existence of a second uracil DNA glycosylase activity in *C. elegans*, its genome does not appear to encode another homolog, (ii) there is no biochemical evidence to show that UNG-1 can process the oxidized base lesion 5hmU, although this is strongly supported by phenotypic data, (iii) it remains uncertain whether UNG-1 and NTH-1 might recognize a broad range of DNA lesions and or serve as sensors to channel the lesions to other DNA repair pathways, and (iv) whether there are distinct complexes involving the DNA glycosylases and AP endonucleases to process oxidative DNA lesions.

We expect that the *C. elegans* model will continue to provide new challenges and uncover novel mechanisms by which this organism uses a limited number of proteins to combat oxidative DNA lesions. It would be interesting to determine whether the *ung-1;nth-1;apn-1;exo-3* quadruple deletion mutant would survive, or other DNA repair pathways would be exploited to repair the accumulated oxidative DNA lesions.

## Author Contributions

NE wrote the entire manuscript. NE and DR revised and edited the final version. Q-MZ-A verified the accuracy of the final version. All authors contributed to the article and approved the submitted version.

## Conflict of Interest

The authors declare that the research was conducted in the absence of any commercial or financial relationships that could be construed as a potential conflict of interest.
